# Effective Biodegradation of Aflatoxin B1 Using the *Bacillus licheniformis* (BL010) Strain

**DOI:** 10.3390/toxins10120497

**Published:** 2018-11-26

**Authors:** Ye Wang, Haiyang Zhang, Hai Yan, Chunhua Yin, Yang Liu, Qianqian Xu, Xiaolu Liu, Zhongbao Zhang

**Affiliations:** 1 School of Chemistry and Biological Engineering, University of Science and Technology Beijing, Beijing 100083, China; yewang6868@126.com (Y.W.); zhanghy@ustb.edu.cn (H.Z.); haiyan@ustb.edu.cn (H.Y.); chyin@sina.com (C.Y.); liuyang@ustb.edu.cn (Y.L.); qianqianxu@ustb.edu.cn (Q.X.); 2 Beijing Agro-Biotechnology Research Center, Beijing Academy of Agriculture and Forestry Sciences, Beijing 100097, China

**Keywords:** aflatoxin B1, biodegradation, induced effect, degradation enzymes, bioinformatic analysis

## Abstract

Aflatoxin B1 (AFB1), a pollutant of agricultural products, has attracted considerable attention in recent years, due to its potential impact on health. In the present study, *Bacillus licheniformis* (BL010) was demonstrated to efficiently degrade AFB1, reducing over 89.1% of the toxin content within 120 h. A crude enzyme solution of BL010 exhibited the highest degradation level (97.3%) after three induction periods. However, uninduced BL010 bacteria was not capable of reducing AFB1. Furthermore, high performance liquid chromatography (HPLC) analysis showed that while a cell-free extract caused a significant decrease in AFB1 content (93.6%, *p* < 0.05), cell culture fluid treatment did not significantly degrade AFB1. The biotransformation products of AFB1 were detected and further identified by quadrupole time-of-flight liquid chromatography–mass spectrometry (LC-Q-TOF/MS); these corresponded to a molecular formula of C_12_H_14_O_4_. A sequence analysis of whole BL010 genes with a bioinformatics approach identified the secondary structures of two degrading enzymes (Chia010 and Lac010), providing an important basis for subsequent homology modeling and functional predictions.

## 1. Introduction

Aflatoxins are a group of secondary metabolites produced predominantly by *Aspergillus flavus*, *A. parasiticus*, and other fungi under certain conditions, and the name aflatoxin has become a generic term for a number of similarly structured chemicals [1,2]. The most toxic compound in this group, aflatoxin B1 (AFB1), has been demonstrated by several studies to have mutagenic, hepatotoxic, teratogenic, and immunosuppressive characteristics [3]. AFB1, which is widely distributed in nature, mainly infects crops, such as peanuts and corn, causing large economic losses every year and potentially harming human and animal health following chronic exposure to even extremely low levels via the food chain [4]. The prevalence of AFB1 is thus considered a severe problem worldwide.

In recent years, the effective removal of AFB1 from food and animal feed has become a hot research topic. Decontamination techniques were reviewed by Zhu et al. [5], and numerous physical and chemical methods have been confirmed to reduce the AFB1 content in crops. However, only a few of these methods conform to the Food and Agriculture Organization’s (FAO) requirement that the reduction of AFB1 is achieved while maintaining the nutritional value without residual toxicity or modifying the food or feed properties [6,7]. Biological degradation has proven to be an efficient, safe, and feasible method for AFB1 removal because its raw materials are non-polluting, highly specific, mild, and environmentally friendly; this process also discourages toxin regeneration [8,9,10].

Various fungi, such as *Dactylium dendroide*, *Rhizopus usarrhizus*, *A. parasiticus*, *Phoma* sp., *Trametes versicolor*, *Armillariella tabescens*, *Aspergillus niger*, and other species can degrade AFB1 to produce degradation enzymes [11,12,13,14,15]. A few studies have also reported the degradation of AFB1 by bacteria, including *Bacillus subtilis* [16], *Mycobacterium fluoranthenivorans* [17], and *Myxococcus fulvus* ANSM068 [7]. In addition, Elsanhoty et al. tested the ability of lactic acid bacteria (*Lactobacillus acidophilus*, *L. rhamnosus*, *L. plantarum*, and *Streptococcus thermophiles*) and probiotic bacteria (*Bifidobacterium angulatum*) to remove AFB1 from contaminated water [18]. The strain TUBF1, belonging to the *Bacillus* sp. group achieved 81.5% AFB1 degradation after 48 h [19].

AFB1 degrading enzymes with active fractions of extra or intracellular metabolites have been isolated from bacteria, fungi, and other microorganisms in a number of studies. An aflatoxin oxidase from *A. tabescens* [20], manganese peroxidase from the white rot fungus *Phanerochaete sordida* YK-624 [21], as well as laccases and chitinases [22,23,24] have been shown to have an AFB1 degrading ability. In addition, whole genome information for BL010 was previously obtained in our laboratory [25] and checked for the presence of any sequences encoding these two enzymes that could be responsible for the previously revealed activity.

There are abundant, diverse microorganism resources in the soil, and recent studies have applied many of these to biodegrade stubborn compounds [26,27]. In a previous study, we isolated and screened a new strain, *Bacillus licheniformis* (BL010), from humid and loose surface soil samples of corn fodder stacks [25]. In the current study, we investigate and explore the degradation and detoxification effects of BL010 on AFB1 liquid cultures and cell-free extract in both induced and uninduced form. We also analyze the characteristics of AFB1 degrading enzymes using bioinformatic methods.

## 2. Results

### 2.1. Biodegradation Kinetics of BL010 in ABS

**Figure 1:**
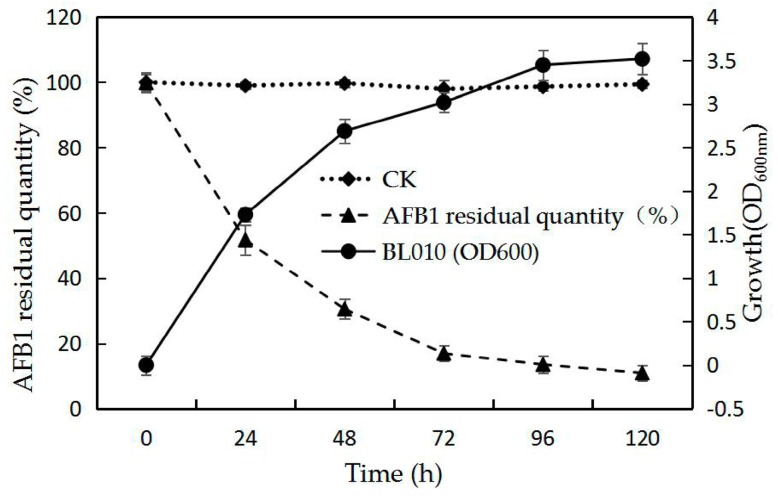
Time course of in vitro kinetics of aflatoxin B1 (AFB1) degradation by *Bacillus licheniformis* (BL010). The initial AFB1 concentration was 50 mg/L; dead BL010 was added to the control group (CK). Values are means of three repeated detections and their standard errors.

The dynamics of BL010 biomass and AFB1 degradation were investigated through experiments performed in ABS medium with BL010 and 50 mg/L AFB1 as the restriction factor (Figure 1). Biomass was evaluated at an optical density of 600 nm (OD_600_) and the AFB1 residual quantity (%) was determined simultaneously (Figure 1). The microbial metabolism was extremely active, with rapid growth from 0 to 48 h, after which growth continued at a slower rate. The AFB1 degradation rate (%) was also highest during this 48 h period; residual AFB1 was only 30.5%, effectively corresponding to OD_600_. After 48 h, the AFB1 degradation rate gradually decreased and became approximately stable as BL010 growth reached stability and declined from 48 to 120 h. The final percentage of AFB1 residue in the culture solution was 10.9%. When dead BL010 was incubated in an AFB1 basic salt solution medium under the same conditions (CK), the final amount of AFB1 residue was not significantly different.

### 2.2. Effect of Induction on Degradation Enzymes

**Figure 2:**
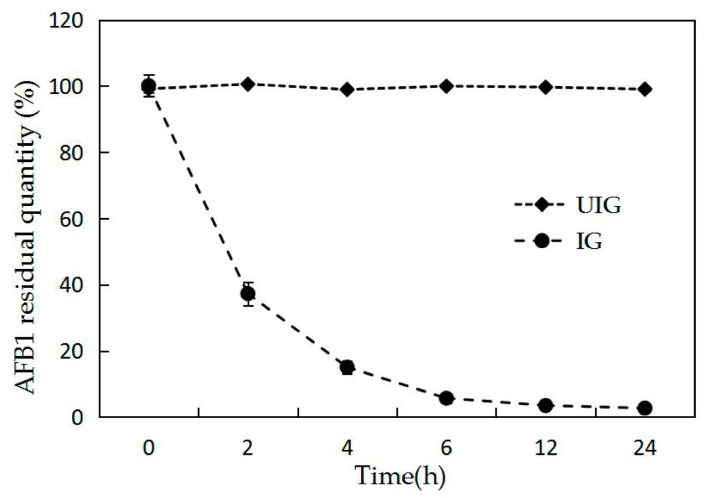
AFB1 (25 mg/L) degradation tests by induced (IG) and uninduced (UIG) enzyme extract, produced by BL010 at 30 °C. Values are means of three repeated detections and their standard errors.

To investigate the features of the BL010 degradation enzyme, we conducted an induction test using 50 mg/L AFB1 as the sole carbon source and an enzymatic degradation test using BL010 from two induced cultures (induced and uninduced) and 25 mg/L AFB1 in crude enzyme solution, as determined by high performance liquid chromatography (HPLC; Figure 2). The AFB1 concentration was stable over a 24 h incubation period in the uninduced crude enzyme solution, and no significant differences were observed between 0 and 24 h; however, the amount of AFB1 in the induced BL010 crude enzyme solution dropped rapidly, and AFB1 was almost completely consumed after 24 h of incubation. The induced enzyme liquid displayed high degradation activity at 4 h, and the AFB1 residual rate was only 15.2%. This may have been due to the restriction on the enzyme amount or the differences between the culture conditions and those of the outside world. As expected, the final residue rate reached 2.7% after 24 h. These results suggest that an AFB1 carbon source is necessary for AFB1 specific degradation enzyme production.

### 2.3. AFB1 Degradation by BL010 Cell Culture Fluid and Cell-Free Extract

**Figure 3:**
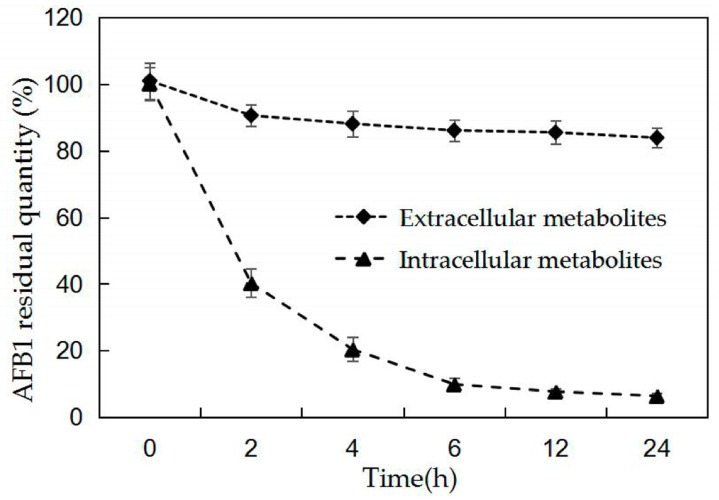
Curves of AFB1 degradation by cell culture fluid (extracellular metabolites) and cell-free extracts (intracellular metabolites) observed over a period of 24 h. Values are means of three repeated detections and their standard errors.

We investigated AFB1 degradation using cell culture fluid and cell-free BL010 extract, induced in ABS three times with AFB1 for 24 h, with an extra incubation using 25 mg/L AFB1 at 30 °C for 24 h. The HPLC analysis showed that AFB1 was relatively stable (11.9% at 4 h and 16.1% at 2 h of AFB1 degradation) over the 24 h incubation period in the extracellular metabolites (Figure 3). A significant level of reduction was observed when AFB1 was treated with intracellular metabolites extracted from broken cells, with a maximum reduction of 79.6% AFB1 at 4 h and 93.6% at 24 h (Figure 3). These results suggest that intracellular metabolites play major roles in AFB1 degradation. In contrast, significant changes in the AFB1 content and shorter degradation periods were observed when intracellular metabolites were used compared to when bacterial cells were used (89.1% AFB1 degradation at 120 h; Figure 1).

### 2.4. AFB1 Degradation Product Analysis

**Figure 4:**
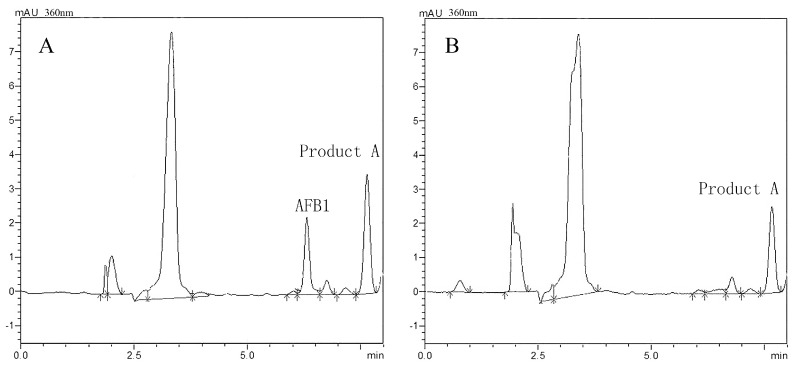
High performance liquid chromatography (HPLC) chromatograms showing AFB1 degradation. (**A**) AFB1 treated with BL010 culture after 48 h of incubation. (**B**) AFB1 treated with BL010 culture after 120 h of incubation.

**Figure 5:**
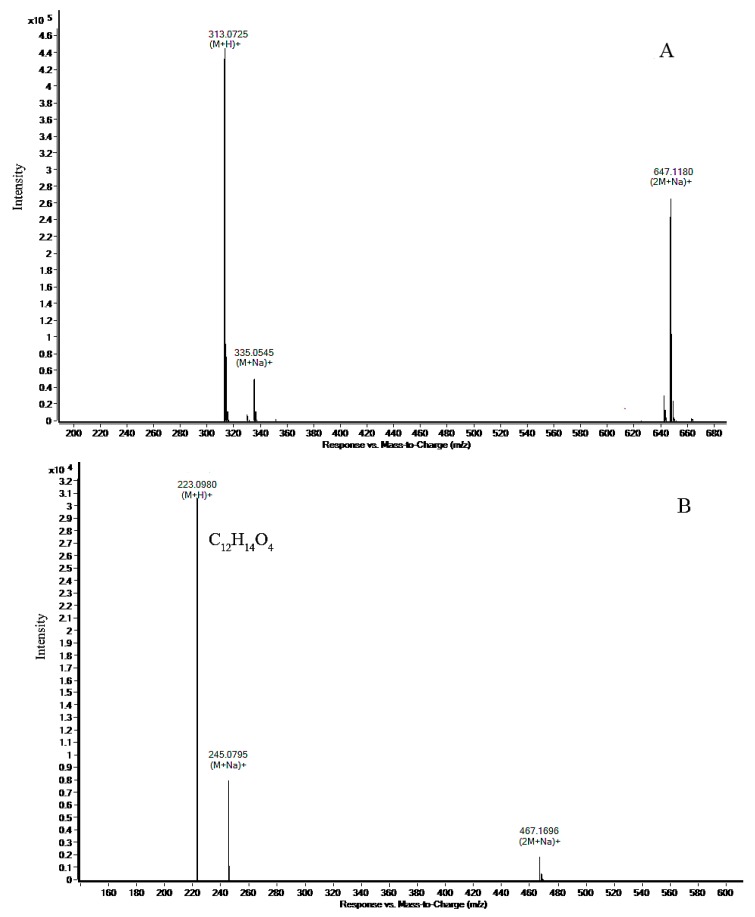
Full mass spectra of AFB1 degradation product A. (**A**) Quadrupole time-of-flight liquid chromatography–mass spectrometry (LC-Q-TOF/MS) spectrum of AFB1 standard. (**B**) LC-Q-TOF/MS spectrum of product A.

AFB1 degradation and its products were simultaneously confirmed by HPLC analysis. Figure 4 and Figure 5 show representative AFB1 chromatograms after 48 and 120 h of incubation in BL010 culture. Figure 4 shows a peak upon the appearance of AFB1 at around 6.5 min, which corresponds to that in Figure S1, and a subsequent peak (product A) at around 7.7 min. AFB1 gradually decreased after 48 h of cultivation (Figure 4A), and product A gradually increased. However, AFB1 was barely detected after 120 h of culture (Figure 4B) and the content of product A was lower than at 48 h. Figure 5A shows the full mass spectrum fragmentation pattern of standard AFB1, exhibiting molecular ions at 313 m/z for [M+H]^+^, 335 m/z for [M+Na]^+^, and 647 m/z for [2M+Na]^+^. These results agree with the corresponding molecular weight of the AFB1 standard molecule with (M+) at m/z = 312. Degradation product A was further identified using quadrupole time-of-flight liquid chromatography–mass spectrometry (LC–Q-TOF/MS) analysis. Ion peaks at m/z = 223, 245, and 467 were the most abundant fragments corresponding to precursor ions at m/z = 313 (see Figure 5B for the molecular formula of C_12_H_14_O_4_).

### 2.5. Genome Identification and Phylogenetic Relationships with Laccase and Chitinase Genes

**Figure 6:**
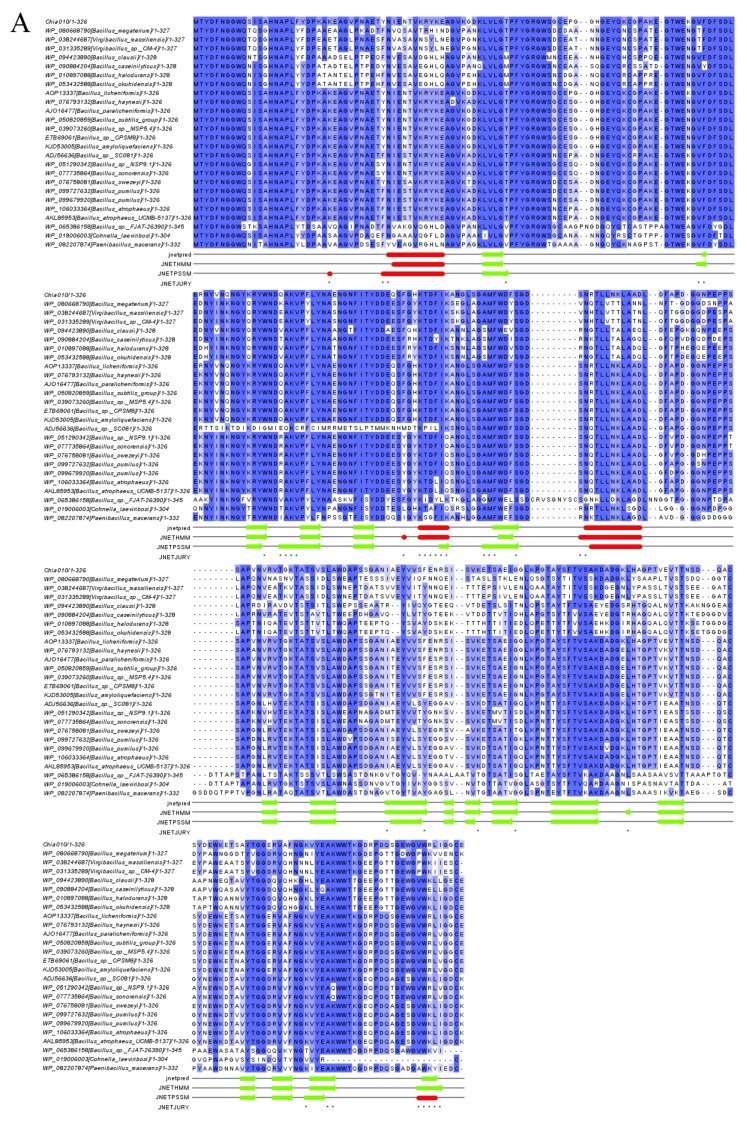

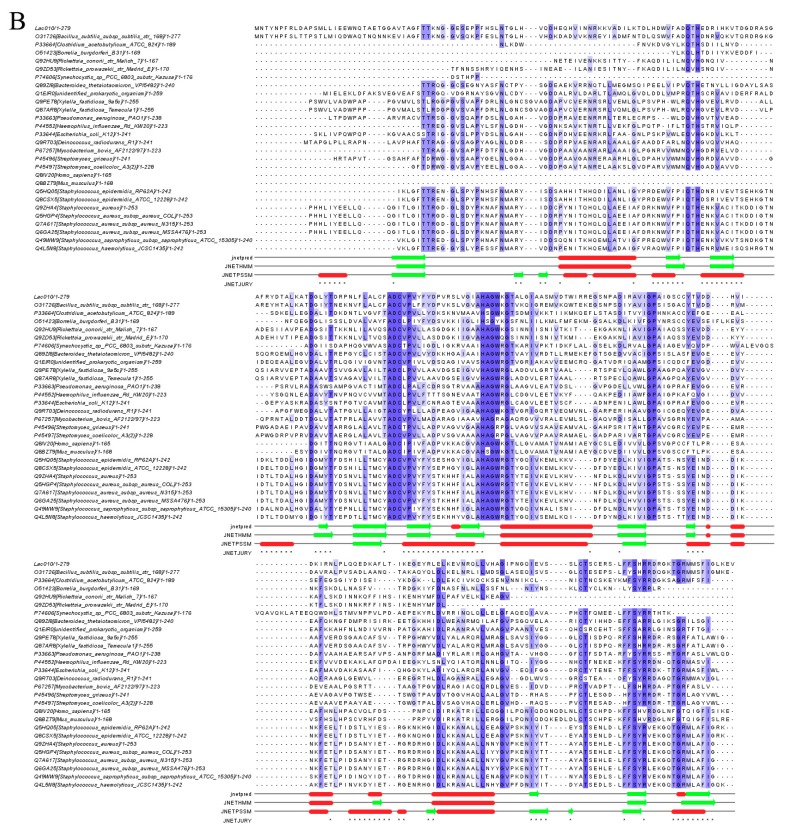
Multiple sequence alignment of the Chia010 (**A**) and Lac010 (**B**) proteins. Highly conserved residues are shown in blue text; darker colors indicate greater conservation. The asterisk (JNETJURY) indicates that all conserved residues in the aligned sequence were completely consistent. Jnetpred is the prediction result, α-helices are indicated by red tubes, and β-sheets are indicated by green arrows. Like JNETPSSM, JNETHMM is a combination of two methods, based on HMM and PSSM.

In this study, laccase and chitinase genes (Lac010 and Chia010) were found in the BL010 genome based on a microbial genome database. Multiple sequence alignments were performed to determine the sequence similarity at the protein level to illustrate conserved and variable sites within Lac010 and Chia010 (Figure 6). The Jalview online server was used for multiple sequence alignments to obtain the conserved sequences of the enzymes and to predict the secondary structures of the two proteins. Conserved regions were found in all aligned laccases and chitinases. The highly conserved residues are shown in Figure 6 in blue text, where darker colors indicate greater conservation. The secondary structures (α-helices and β-sheets) of the enzymes were also determined. It was shown that Chia010 contains three α-helices and 18 β-sheets (Figure 6A), and Lac010 contains nine α-helices and 12 β-sheets (Figure 6B).

**Figure 7:**
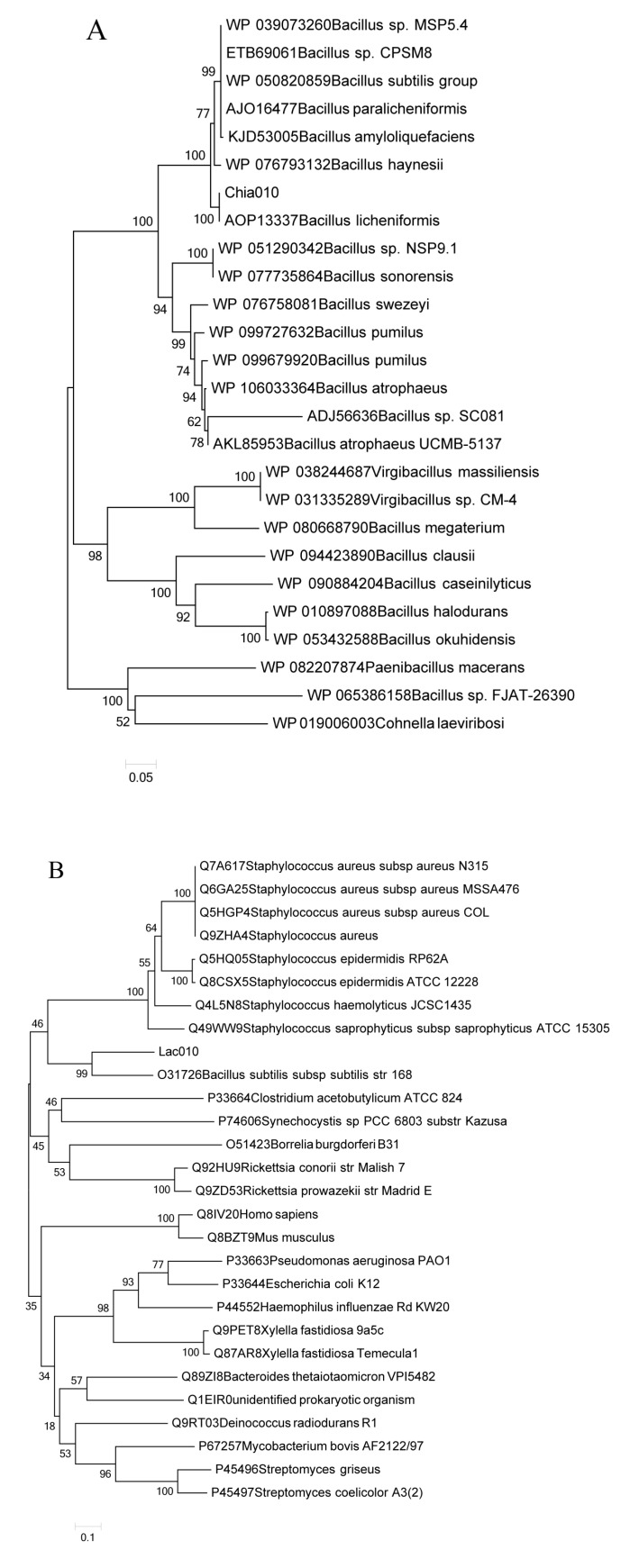
Phylogenetic tree analysis of Chia010 (**A**) and Lac010 (**B**) from BL010. The tree was calculated with p-distances using the MEGA v.6.06 software, based on a Clustal W alignment. Bootstrap values per 100 bootstrap analyses are indicated on the tree. The scale bars indicate distances equivalent to 0.05 (**A**) and 0.1 (**B**) amino acid substitutions per site.

Using the alignments of multiple amino acid sequences, two phylogenetic trees were constructed to further show that laccase and chitinase have a distinct clade that differs from those of the previously determined laccases and chitinases of other microorganisms (Figure 7). Figure 7A indicates that Chia010 and other chitinases (from *B. licheniformis*, *B. haynesii*, *B.* sp., and *B. subtillis* groups) evolved from a common ancestor. The genetic distance between Chia010 and *B. licheniformis* chitinase is relatively short, with *Bacillus* sp. FJAT-26390 having the most distant relationship. Figure 7B shows that the genetic distance between Lac010 and *B. subtilis* subsp. str. 168 laccase is relatively short, with *Escherichia coli* K12 having the most distant relationship.

## 3. Discussion

AFB1 is a chemically stable mycotoxin that is distributed worldwide. It represents an economic challenge to food and animal feed industries and a health hazard to consumers. AFB1 must be treated using microbes to achieve complete degradation, and the identification of these beneficial microbes is an active field of research [28,29]. In the present study, a BL010 biomass was observed in medium containing AFB1 (Figure 1), and the dynamics of microbial and AFB1 degradation were investigated. When the growth rate was delayed, the rate of toxin degradation reduced, suggesting that BL010 was able to remove AFB1 by using it as a carbon source for its own growth. This ability has also been confirmed for other microorganisms [30]. Previous studies have tested the impacts of living bacteria belonging to *Bacillus* (Gram-positive) classes on AFB1 degradation and reported that cell-free supernatant fluid of *B. subtilis* UTBSP1 (78.39%) [31] and *B.* sp. (2) (84.8%) [32] has the capacity to degrade AFB1. The degradation of AFB1 when co-incubated with BL010 in the current study was much higher, whereas the toxin content in dead cells remained virtually unchanged (Figure 1). The corn feed that produced the AFB1 used in this study may have been polluted by aflatoxigenic fungus, such that the high AFB1 concentration became a selection pressure, giving this strain an advantage over other strains [2,33]. Wu et al. showed that the mechanism of aflatoxin reduction by certain probiotics relied on the latter’s adhesion to cell wall components [1]. The reduction of the AFB1 concentration in the BL010 culture was clearly not caused by a cell adhesion mechanism; instead, it primarily occurred through biodegradation by living cells.

The degradation reaction did not occur rapidly in previous studies, but rather, began with a delay ascribed to the synthesis of enzymes responsible for degradation, which was assumed to be induced by the toxin; this ability has also been confirmed for other microorganisms [34]. As shown in Figure 2, the crude enzyme of uninduced BL010 was incapable of degrading the toxin. The inducible character of this phenomenon was further proven by the removal of AFB1 in the presence of the crude enzyme of induced BL010 [35]. To determine the role of enzyme functional systems in AFB1 degradation, the degradation ability of the culture supernatant and cell-free extracts was tested [36] (Figure 3). According to the HPLC analysis, there was no significant difference in the aflatoxin conversion rates in the cell culture fluid within 24 h, and AFB1 degradation reached a maximum in the intracellular metabolites. Similarly, a reduction in AFB1 was observed in the presence of *Rhodococcus* cell-free extracts [37], implying that the intracellular metabolites of BL010 play major roles in AFB1 degradation. Conversely, our results regarding the cell-free extract of BL010 were different from those reported by Wang et al. [30] and Teniola et al. [14]. This discrepancy was likely due to the major role played by enzymes in the TADC7 culture broth [30] and the culture broths of the four bacterial strains used in AFB1 degradation in these previous studies [14].

One intermediate appeared during the BL010 degradation of AFB1, and AFB1 finally disappeared after treatment for 120 h with BL010 culture (Figure 4). These observations, which were made using HPLC quantification techniques (Figure 1), confirmed the degradation of AFB1 by BL010. Near elimination of AFB1 was previously reported by Sangare et al. in culture with the *P. aeruginosa* isolate N17-1 after a 120-h incubation period [38]. The HPLC results of the present study showed a clear peak for product A, appearing at around 7.7 min. The amount of product A was less after cultivation for 120 h than that at 48 h, possibly because after BL010 had consumed the AFB1, product A was used as a secondary carbon source for growth. We identified the 223 m/z product from the AFB1 biodegradation sample by LC–Q-TOF/MS analysis and found that it corresponded to a molecular formula of C_12_H_14_O_4_ (Figure 5). However, it is difficult to speculate on the molecular structure of product A, because the obtained product content was very low, and the purification process is difficult. The metabolization of AFB1 to other unknown compounds that can be detected by HPLC and/or LC–MS has been reported previously [1,5,8,28,39,40]. There have also been reports of significant AFB1 reduction, but no product has been detected [38,41,42]. These results suggest that AFB1 is likely metabolized to degradation products with chemical properties that differ from those of the parent compound [43], perhaps because this process is catalyzed by multiple enzymes [42].

The entire BL010 genome and bioinformatics analysis identified the sequences of degradation enzymes Chia010 and Lac010. The amino acid sequences of these proteins showed the highest similarity with chitinase from *B. licheniformis* (100%) and laccase from *B. subtilis* subsp. str. 168 (99%; Figure 7). This high sequence similarity indicated a close relationship between the proteins and facilitated recognition of the similarity among protein secondary structures [44]. We performed multiple sequence alignments on all sequences involved in phylogenetic tree construction. Figure 6 shows the Chia010 and Lac010 protein sequence alignments and their structural relationship. Chia010 was relatively conserved during the process of evolution. The structure of Chia010 was dominated by β-sheets (Figure 6A), while of Lac010 was dominated by α-helices (Figure 6B). Lac010 showed fewer conserved regions, suggesting that there were some differences in their evolution processes. Future studies should predict the secondary structures of these enzymes using multiple sequence alignment to provide an important basis for homology modeling. Secondary structure predictions will also contribute to the identification of the functional domains of these enzymes [45]. However, the potential involvement of Chia010 and Lac010 in the revealed activity requires subsequent experimental confirmation.

## 4. Conclusions

In summary, *B. licheniformis* (BL010), which was obtained during our previous study, was observed to degrade AFB1. The strain exhibited high AFB1 degradation activity with initial concentrations as high as 50 mg/L, suggesting that it is an efficient AFB1 degrading strain. An increase in the degradation activity of degradation enzymes was the result of induction by a high concentration of AFB1. We found and proved that the cell-free extract of BL010 plays a major role in the degradation of AFB1, as well as product A. Thus, we proposed the use of bacterial strain BL010 for the detoxification of AFB1. We also found two degradation enzyme genes using a bioinformatics approach and constructed the phylogenetic tree and multiple sequence alignments. Sequence analysis determined the secondary structures of the two enzymes, which provides an important basis for subsequent homology modeling and functional predictions.

## 5. Materials and Methods

### 5.1. Reagents and Materials

Standard AFB1 was purchased from Bailingwei Technology Co. Ltd. (Beijing, China) at >99.9% purity. As ethanol can dissolve AFB1 [28], solid AFB1 was diluted with ethanol to prepare stock solutions of 500 mg/L (4 °C). All chemicals used were of analytical grade. HPLC grade solvents and the HPLC pump (model LC-10AT) were purchased from Shimadzu (China). We used a Diamonsil C18 column (4.6 mm × 250 mm, 5 μm; Dima Technologies, Beijing, China).

### 5.2. Strain and Media

BL010 was obtained for our previous study and cultivated on different media: LB medium was used as the basic and enrichment medium [40], and the basic salt solution medium was used as the domestication and degradation medium. This consisted of 0.5 g/L NH_4_Cl, 0.5 g/L KH_2_PO_4_, 0.1 g/L MgSO_4_, 0.01 g/L CaCl_2_, 0.01 g/L ammonium ferric citrate, and 0.5 mL/L trace element solution (0.22 g/L FeSO_4_•7H_2_O, 0.85 g/L Co (NO_3_)_2_, 0.83 g/L NH_4_MoO_4_, 3.96 g/L MnCl_2_•4H_2_O, 5.0 g/L CuSO_4_•5H_2_O, 5.74 g/L ZnSO_4_•7H_2_O, and 1.24 g/L H_3_BO_3_. The pH of the medium was adjusted to 6.8–7.0 using Na_2_CO_3_ (ABS). To prepare the 50 mg/L ABS, AFB1 ethanol stock solution was blown dry from a 100 mL conical flask using a blower. The basic salt solution medium was then added to the conical flask and mixed by ultrasonic shaking for 30 min. All suspensions were sealed with a sterilized filter membrane and autoclaved for 30 min at 121 °C and 0.1 MPa. The main phosphate buffered saline (PBS) ingredients were 80 g NaCl, 0.2 g KCl, 1.42 g Na_2_HPO_4_, 0.27 g KH_2_PO_4_, 800 mL distilled water; the pH was adjusted to 7.0 using NaOH or HCl, and distilled water was added to an initial volume of 1 L.

### 5.3. Biodegradation of AFB1 in ABS

The BL010 strain in the glycerol tube was immediately transported to LB liquid medium and activated at 30 °C for 48 h. The activation process was repeated at least three times in the same LB liquid medium to increase the number of bacteria. Biodegradation studies were conducted in a series of 50 mL sterile glass flasks. We inoculated 100 μL of the third activated strain into ABS (1:50, *v*/*v*) to give a final AFB1 concentration of 50 mg/L and subsequently, incubated the mixture at 30 °C on a rotary shaker at 200 rpm for 120 h; induction was performed as described in Section 5.4. The basic salt solution medium with dead BL010 was cultivated under the same conditions for use as a control group (CK). After induction, the ABS culture fluid was centrifuged to discard the supernatant, and the precipitate was then washed three times with the basic salt solution medium. AFB1 (50 mg/L) was added to the basic salt solution medium for culture. Samples were then cultivated for 0, 24, 48, 72, 96, or 120 h. At each time point, one experimental sample of 200 μL culture fluid was used. Each 200 μL culture fluid sample was added to an equal volume of ethanol to terminate the degradation reaction, and all liquid was passed through a sterile 0.22 μM pore filter (Agilent Technologies, Santa Clara, CA, USA) to remove impurities, such as cells, and the resulting liquid was retained for further experiments. All processed samples were preserved at –25 °C until the HPLC analysis.

### 5.4. Effect of Induction on Degradation Enzymes

To explore the effect of induction on degradation enzymes, one BL010 bacterial sample was cultivated to the logarithmic growth phase in LB medium (uninduced). The biomass was then evaluated in terms of OD_600nm_ [30]. Another sample of 1 mL LB culture fluid was transferred to the ABS medium (induction medium) and induction culture (1:50, *v*/*v*) to give a final AFB1 concentration of 50 mg/L for 24 h. The induction process was repeated three times (induced). We added 20 mL of each of the two different bacterial suspensions (induced and uninduced) to an ice bath for ultrasonic disruption of the cells. The instrument parameters were as follows: Power, 400 w; working time, 5 s; interval time, 5 s; and effective crushing time, 15 min. The crude enzyme solution was obtained by centrifugation at 10,000 rpm for 10 min, and this was passed through a 0.22 μM pore filter to remove cell debris. Subsequent processing of the AFB1 degradation system was performed as follows: 400 μL crude enzyme solution and 1.6 mL PBS was added to a polypropylene centrifuge tube that contained dried AFB1 particles, to give a final volume of 2 mL. The initial AFB1 concentration was 25 mg/L (2:25, *v*/*v*). All mixtures were reacted at 30 °C on a rotary shaker at 200 rpm/min for 0, 2, 4, 6, 12, or 24 h, and 200 μL aliquots were sampled from the mixtures at each time point. The completed samples were then subjected to the filtration and instrumental analysis procedures described in Section 5.3 and Section 5.6.

### 5.5. Biodegradation Activity Test of BL010 Cell Culture Fluid and Cell-Free Extract

As described in Section 5.4, BL010 strains were cultivated in 50 mg/L AFB1 and the basic salt solution medium (1:50, *v*/*v*) at 30 °C for 24 h; this procedure was repeated three times. This induction test method was used to increase the sample’s detoxification ability. As part of the sampling process, cells were harvested by centrifugation (8000 rpm, 10 min, 4 °C), and cell culture fluids (extracellular metabolites) were separated. Harvested cell precipitate was washed with 20 mL PBS (pH = 7.0); this procedure was repeated three times, and cells were then mixed with 20 mL PBS in an ice bath for ultrasonic cell disruption. Finally, cell-free extracts (intracellular metabolites) were obtained. Processing and detection of the 25 mg/L (2:25/v:v) AFB1 degradation medium by extracellular and intracellular metabolites were performed as described above.

### 5.6. Analysis of Aflatoxin B1 Concentration and Degradation Products

The AFB1 degradation ability was confirmed using an HPLC system with an ultraviolet (UV) diode array detector at 360 nm; the specific detection conditions are listed in Table 1. LC-Q-TOF/MS (Agilent) was applied to analyze the AFB1 degradation products. All samples were filtered through a sterile 0.22 μM pore filter (Agilent) before testing.

### 5.7. Identification and Bioinformatics Analysis of BL010 Degradation Enzymes Genes

We applied a bioinformatics approach, in which BLASTp was performed using the protein coding sequence of bacteria laccases and chitinases as a query against the BL010 genome database. Whole genome information for BL010 had been previously obtained in our laboratory [25]. To determine the evolutionary relationship between the two BL010 bacterial enzymes and other laccases and chitinases, we performed a phylogenetic analysis using the amino acid sequences of BL010 laccase, 27 other laccases, chitinase, and 25 other chitinases obtained from the National Center for Biotechnology Information (NCBI) database using neighbor joining methods with 1000 bootstrap replications performed by Molecular Evolutionary Genetics Analysis (MEGA) software v.6.06 [46]. Amino acid sequences encoded by laccase and chitinase genes in the BL010 genome were determined based on a Clustal W alignment [47], and a phylogenetic tree of the two enzymes’ genes was then produced. Multiple sequence alignments were constructed using the Jalview multiple sequence and structure alignment online server.

### 5.8. Statistical Analysis

All experiments were performed in triplicate. Data were analyzed in a completely randomized single factor design by analysis of variance (ANOVA) using the general linear model procedure in the SAS statistical software. Statistical significance was set at a level of *p* < 0.05. When a significant F-value was detected, we used Duncan’s multiple range test to determine significant differences among means.

## Figures and Tables

**Table 1:** HPLC detection conditions for AFB1 in samples.

Parameter	Condition
Column	Diamonsil C18 5 μm (250 × 4.6 mm)
Mobile phase	Acetonitrile (solvent B)/water (solvent A) = 45:55 (*v*/*v*)
Velocity	1 mL/min
Injection volume	20 μL
Detection wavelength	360 nm
Aflatoxin B1 Retention time	6.5 min
Product A Retention time	7.7 min

## Supplementary Materials

The following are available online at http://www.mdpi.com/2072-6651/10/12/497/s1. Figure S1: Ultraviolet scans and chromatograms of AFB1 obtained by HPLC, which showed that the maximum absorption peak of AFB1 was around 360 nm.
