# Supplementary material for: Effective Biodegradation of Aflatoxin B1 Using the Bacillus licheniformis (BL010) Strain
Source: Toxins (Basel). 2018 Nov 26;10(12):497. doi: 10.3390/toxins10120497 (PMC6315853; doi:10.3390/toxins10120497)
Supplement: Supplementary file 1 [file toxins-10-00497-s001.pdf]

Supplementary Materials: Effective Biodegradation of Aflatoxin B1 Using the Bacillus licheniformis (BL010) Strain

Ye Wang, Haiyang Zhang, Hai Yan, Chunhua Yin, Yang Liu, Qianqian Xu, Xiaolu Liu and **Zhongbao Zhang**

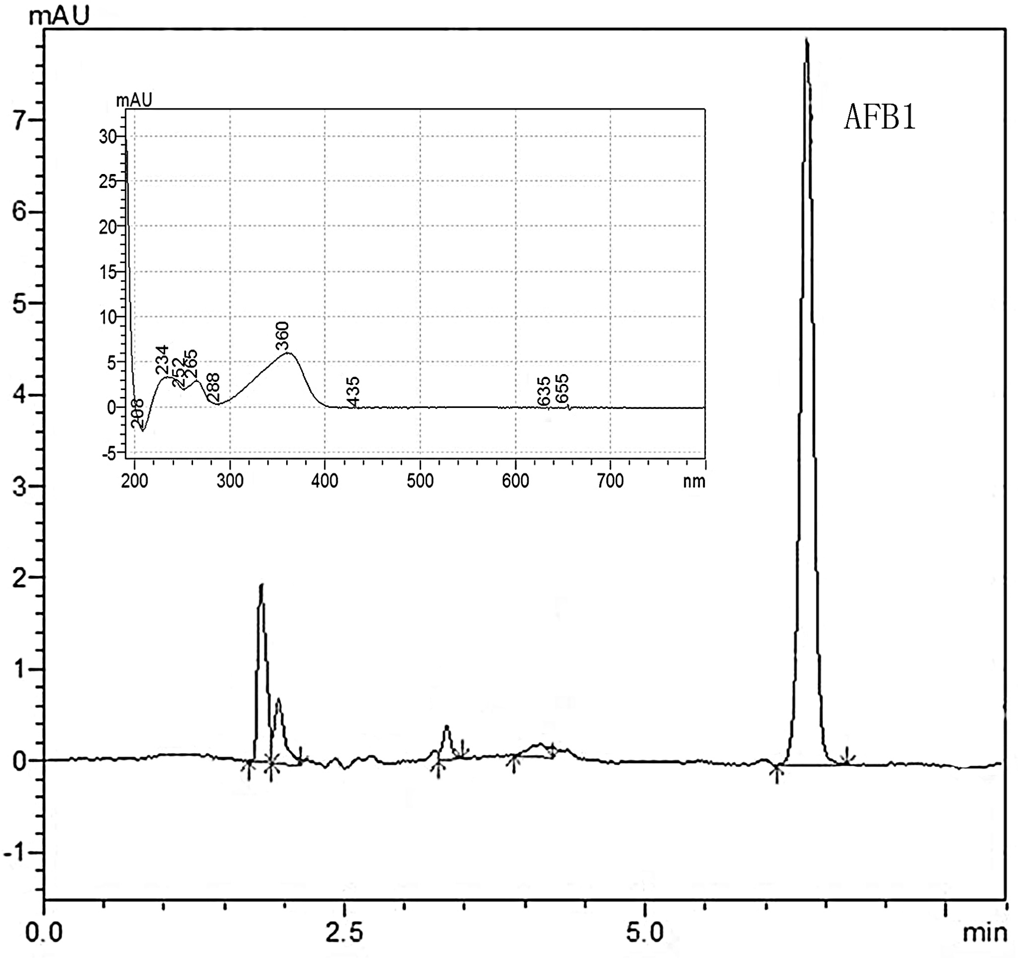

Figure S1. Ultraviolet scans and chromatograms of AFB1 obtained by HPLC, which shown that the maximum absorption peak of AFB1 was around 360 nm.
